# Knowledge and risk perceptions of Israelis towards combustible cigarettes: the need for immediate remedial action

**DOI:** 10.1186/s13584-018-0276-2

**Published:** 2019-01-14

**Authors:** Laura J. Rosen, David A. Rier, Robert Schwartz, Michal Talitman, Lior Zwanziger

**Affiliations:** 1 Department of Health Promotion, School of Public Health, Sackler Faculty of Medicine, Tel Aviv University, POB 39040, 69978 Ramat Aviv, Israel; 2 Center for Global Tobacco Control, Department of Social and Behavioral Sciences, Harvard T. H. Chan School of Public Health, 677 Huntington Ave, Boston, MA 02115 USA; 3 Department of Sociology and Anthropology, Bar Ilan University, 5290002 Ramat-Gan, Israel; 4 University of Toronto, Toronto, M5S 1A1 Canada; 5 Department of Statistics, Tel Aviv University, 69978 Ramat Aviv, Israel; 6 Sackler Medical School, Tel Aviv University, 69978 Ramat Aviv, Israel

**Keywords:** Tobacco, Smoking, Secondhand smoke, Risk perceptions, Knowledge

## Abstract

**Background:**

Devastation from the tobacco epidemic continues, with strong government tobacco control policy absent in most countries. Knowledge of the full scope of tobacco harm in populations may form the basis for healthier behavior, de-normalization of smoking, and a consensus about necessary public policy. However, many populations may be poorly-informed about the risks, and this ignorance may undermine both effective policy-making and implementation of tobacco control policies. We present knowledge and risk perceptions about smoking tobacco smoke exposure in Israel.

**Methods:**

A nationally-representative phone survey was conducted in Israel (*n* = 505; response rate = 61%). We assessed knowledge about active and passive smoking using four questions, three of which addressed knowledge about harm, and one of which addressed knowledge of tobacco-related harm relative to knowledge of harm due to traffic accidents. The three questions which addressed knowledge of harm were combined into a composite score. We also asked four risk perception questions concerning tobacco smoke exposure, which were measured on a 7-point Likert scale and then combined. Multivariable logistic regression and linear models were used to identify whether smoking status or socio-demographic variables were associated with knowledge of harm, comparative knowledge of harm, and risk perceptions.

**Results:**

Just two in five respondents, and one in five respondents who were current smokers, accurately answered three simple questions about harms of smoking. Fewer than three in ten respondents, and fewer than one in five smokers, knew that smoking causes more damage than traffic accidents. Many (30.3%) were unaware that tobacco smoke exposure causes both lung cancer and heart disease, 27.7% did not know that smoking both shortens life and injures quality of life, and 31.1% did not know that smoking-attributable health problems will afflict all or most heavy smokers. Overall, risk perceptions regarding tobacco smoke exposure were high (mean = 24.5, SD:4.5, on a scale of 7–28, with 28 the indicating highest level). Smoking status was consistently associated with lower levels of knowledge, comparative knowledge, and risk perceptions, with current smokers having the lowest levels of knowledge and the lowest risk perceptions.

**Conclusions:**

Like many others, Israelis, and particularly Israeli smokers, do not fully grasp tobacco’s true dangers. Effective communication of the full range of tobacco risks to the public, with a focus on communication with smokers, is an essential component of comprehensive tobacco control policy.

**Electronic supplementary material:**

The online version of this article (10.1186/s13584-018-0276-2) contains supplementary material, which is available to authorized users.

## Introduction

The tobacco epidemic continues with its devastating toll, despite decades of research and extensive knowledge about the consequences of tobacco use [1]. World-wide, effective governmental policies are regularly blocked by tobacco industry lobbying [2]. Partial or unenforced advertising bans permit the tobacco industry to continue to lure young smokers into life-long tobacco addiction. Millions of non-smokers are regularly exposed to tobacco smoke [3], putting them at inadvertent risk, due to incomplete or poorly-enforced laws, or exposure in private places. Permanent smoking cessation is an elusive goal for many smokers, even when aided by first-line pharmaceutical and counseling approaches [4].

Dissemination of information of risks regarding tobacco products has an important influence on population knowledge and related behavior. For example, anti-smoking media campaigns have played an important role in influencing the public around the world. The *Tips from a Former Smoker* campaign in the US, which cost the Federal government $54 million in 2012 [5], was found to increase both knowledge and risk perception [6], and ultimately led to an estimated 1.6 million Americans attempting to quit smoking [7]. There was a similar effect on knowledge following a media campaign, accompanied by health warning labels, in Mexico [8]. The impressive level of knowledge among Canadians has been attributed to the high-budget anti-smoking educational program established by their government, which designated $480 million for an anti-smoking campaign, of which 40% was dedicated to mass media campaigns [9].

In contrast to policies in these countries, there has never been public funding for ongoing education of the Israeli public about tobacco products: public education campaigns are rare, limited in scope, and generally not funded by the government. Of note is the work done by the Israel Cancer Association, which has funded various campaigns over the years. Smoking prevalence declined in Israel for decades [10], but that decline has ceased in the past decade [11], with rising smoking rates among young adults recently reported [12]. This may be related to low levels of knowledge of harms due to smoking, as well as failure to update and extend once-strong tobacco-control policies [13]. Cigarette packs include the same small, text-only messages written over a decade ago, and Israel has not yet mandated graphic warnings. The tobacco industry, conversely, invests tens of millions of dollars annually in advertising and promotion of its products [10]. The public may thus be misinformed about the full scope of risks; however, monitoring of knowledge of tobacco-related harm and risk perceptions associated with smoking is not conducted systematically.

Some years ago, we conducted a nationally-representative study of Israeli adults regarding tobacco-related behaviors, attitudes, and beliefs. We previously reported on public support for smoke-free areas [14, 15]. The current paper presents data on knowledge and risk perceptions regarding use of and exposure to combustible cigarettes (CCs) among Israeli adults, by smoking status, and identifies correlates associated with knowledge and risk perceptions.

## Methods

### Study design and sampling strategy

The details of the study design and sampling strategy have been reported previously [14, 15]. In brief, a national phone survey of Israeli adults was conducted in Israel by the Cohen Survey Institute at the end of 2010. The survey was representative of adults aged 18 and above living in private residences with land telephone lines. Sampling was done in stages, according to statistical areas (randomly sampled within constructed layers), residences within statistical areas (simple random sampling), and individuals within residences (last-birthday method).

Questionnaires were translated by professionals in the Cohen Institute. Respondents chose whether to complete the survey in Hebrew, Arabic, or Russian.

The planned sample size of 500 was calculated to allow sufficient precision in estimating the study’s primary endpoint: public support for smoke-free air, which was previously reported [14]. In order to obtain a 60% response rate with a final sample size of 500, 1077 phone numbers were selected for inclusion.

### Ethical approval

The study was approved by the Tel Aviv University Ethics Committee in April, 2008.

### Questionnaire and variables

All relevant questions can be found in Additional file 1.

#### Socio-demographic variables

Population sector was determined from questions about religion, birthplace, and language of interview. All those who identified as Jews were considered part of the Jewish sector. All who identified as Muslim or Druze were considered part of the Arab sector. Those who identified as Christians were categorized into population sector according to birthplace and language of interview. Those who did not specify a religion, but who answered in Arabic, were considered part of the Arab sector.

Age, sex, education, and family income level were asked using a standard set of questions used by the Cohen Institute. We categorized age into four categories: 18–29; 30–49; 50–65; over 65. Information was asked about highest educational institution at which the respondent studied. We collapsed the possible six options into three categories: < 12 years, 12 years, > 12 years.

We asked about income level using two questions. The first referred to family economic status (How would you rate the financial status of your family?), and had five possible levels: very high, high, moderate, low, very low. The second asked about family income. It began with the statement, “The average family income in Israel today is about NIS 11,000” and asked whether family income was much above average, above average, equal, less than average, or much below average. Because we expected (and found) correlations between these two questions, we opted to include just one of the questions in our main analyses. The question on family financial status was chosen for the main analyses because: 1- financial status in Israel, as in many other countries, is not solely a function of monthly income; and, 2 – there were numerous missing values on the measure of family income. A sensitivity analysis was performed using the family income variable.

#### Smoking status

Smoking status referred to use of combustible cigarettes, and was originally asked using five categories (Regular smoker, Occasional smoker, Former regular smoker, Former occasional smoker, Never smoker). We recoded these to three categories (Current smoker, Former smoker, Never smoker).

#### Knowledge and risk perceptions

We used previous questions and questionnaires to inform our questions about knowledge and risk perceptions [16, 17], including some developed and validated by authors (LJR, DR, RS) of this paper [14, 18]. Most of the questions were originally written in English or taken from English publications and translated to Hebrew.

Four knowledge questions were asked. Three of them concerned harm due to smoking, and are referred to in this paper as “knowledge of harm”. The first was about the effect of smoking on life expectancy, the second about whether the effect of ongoing exposure to passive smoke increases the risk of lung and heart disease, and the third was about whether smoking causes general health problems. We also asked one comparative harm question: whether the number of smoking-attributable deaths was greater, equal, or less than the number of deaths attributable to traffic accidents. This is referred to in this paper as “comparative harm”.

The exact questions, and answers, are available in Additional file 1.

In order to examine risk perceptions regarding secondhand smoke, we used questions relating both to likelihood of harm and severity of harm, as per the Health Belief Model [19], and in continuation of our previous work on risk perception [18]. Four questions were asked on a 1–7 point Likert Scale. The first question asked the likelihood that a child exposed to smoking in a car will be harmed. The second question queried the likelihood that an adult exposed to secondhand smoking will be harmed. For these two questions, 1 represented low likelihood and 7 indicated high likelihood. The third question asked about the severity of the damage to a child travelling in a car with people smoking. The fourth question queried the severity of the damage to an adult exposed regularly to secondhand smoking. For these two questions, 1 represented minimal damage and 7 meant severe damage. Those who did not provide an answer due to refusal or because they did not know the answer were coded as “9”, which was considered missing in all analyses.

#### Composite scores

The Composite Knowledge Score (CKS) combined responses to three questions covering basic knowledge of the effects of smoking. Respondents choosing the correct option received one point in their CKS for each correct answer. Incorrect answers garnered zero points in their CKS. Those answering, “Don’t know” to a particular question were included and given a zero for that question. Those refusing to answer were excluded from the analysis. The maximum total score was three, and the minimum was zero.

In cases where the respondents had answered all three questions, we created a binary knowledge summary variable (CKS-BIN) based on answering the three questions assessing knowledge of harm correctly (Yes/No). Those respondents who answered all three questions correctly received a value of one and those who answered all the questions but didn’t answer them all correctly received a value of zero. All other respondents received a value of “missing”.

The Composite Risk Perception Score (CRPS) examined the participant’s perception of risk, and was based on summing the four risk perception questions. Those who did not provide an answer to any of these four questions were excluded. The maximum total CRPS score was 28, and the minimum was four. We calculated Cronbach’s alpha for CRPS.

#### Validation

As previously reported, in addition to the initial validation done on the risk perception questions prior to the conduct of this survey [18], the entire questionnaire was validated in Hebrew. All questions were piloted on a separate sample of 15 individuals. Then, the questionnaire was validated using a test–retest approach, with an interval of one week, with 20 individuals. The final questionnaire was professionally translated from Hebrew to Arabic and Russian.

### Statistical analyses

We calculated percent of respondents who correctly answered each of the three knowledge questions, and the CKS-BIN, by smoking status and overall. We examined differences in CKS-BIN by population sector, gender, age category, and smoking status using the Chi-squared test. We used logistic regression to perform univariable analyses on educational level and family financial status, to account for the continuous nature of those variables. We used multivariable logistic regression to examine the influence of the same set of variables on CKS-BIN. We present the adjusted odds ratios (ORs) with confidence intervals (CIs) and *p*-values.

We present means, standard deviations, and n’s of CRPS by smoking status and levels of possible explanatory variables for the risk perception questions. One-way analysis of variance was used to test for differences in CRPS by smoking status. We used multivariable analysis of variance to examine the influence of population sector, gender, age category, smoking status, educational level, and family financial status on CRPS. We present the Least Squared Means and *p*-values.

For all analyses, socio-economic status [SES] was based on reported family financial status. We also performed a sensitivity analysis using reported family income instead of family financial status in the multivariable models.

The analyses were conducted using the statistical programs R and SAS Version 9.4.

## Results

### Study sample

The total sample size was 505. In order to achieve this with a response rate of at least 60%, 1077 numbers were originally selected for inclusion. After excluding 168 numbers which were disconnected, 47 faxes or modems, and 33 businesses, 829 phone numbers were available. Full interviews were obtained from 505, giving a response rate of 61%. Among non-respondents, 23% were refusals, 10% didn’t answer, 5% were excluded due to communication problems (individuals didn’t speak Hebrew, Russian, or Arabic, or had difficulties in hearing or understanding), and 1% were partial interviews.

There were 420 respondents categorized to the Jewish sector (417 Jews, one Christian, and 2 USSR-born respondents who did not specify a religion) and 84 respondents to the Arab sector (61 Muslims, 8 Druze, 12 Israeli-born Christians, one Lebanese-born Christian, and two Israeli-born respondents who did not specify a religion and who answered in Arabic). Population sector could not be determined for 1 participant.

Of the 505 respondents, about half were male and half female (females: 50.7%, males: 49.3%). About half (52.3%) had at least some post-high school education. Most (62.3%) reported their family’s financial status as average, while 21.7% reported above-average income and 16.0% reported below-average income. Current smokers comprised 22.3% of the sample, 26.9% were former smokers, and 50.8% were never smokers.

Distributions of nationality (Jews/Non-Jews), educational level, and gender were similar to those reported by the Central Bureau of Statistics [14]. The percentage of current smokers was very close to that reported in the 2010 Health Ministry report (23.3%) [20].

Distributions of all variables by smoking status are presented in Table 1.

**Table 1:** Smoking status (Current, Former, Never) of study participants, by levels of demographic variables

		Current Smoker % (*N* = 112)	Former Smoker % (*N* = 135)	Never Smoker % (*N* = 255)	Overall % (*N* = 502)	*P*-value
Overall (*N* = 502)		22.3	26.9	50.8		
Population Sector (*N* = 501)	Jewish (*N* = 417)	21.8	28.5	49.6	83.2	0.202
	Arab (*N* = 84)	25.0	19.0	56.0	16.8	
Gender (*N* = 502)	Male (*N* = 248)	29.4	34.3	36.3	49.4	< 0.001
	Female (*N* = 254)	15.4	19.7	65.0	50.6	
Age group (*N* = 496)	18–29 (*N* = 88)	17.1	22.7	60.2	17.7	< 0.001
	30–49 (*N* = 185)	31.4	16.2	52.4	37.3	
	50–65 (*N* = 148)	19.6	37.2	43.2	29.8	
	> 65 (*N* = 75)	12.0	38.7	49.3	15.1	
Educational level (*N* = 498)	< 12 (*N* = 48)	29.2	39.6	31.3	9.6	0.002
	12 *N* = 189)	28.0	21.7	50.3	38.0	
	> 12 (*N* = 261)	16.5	28.7	54.8	52.4	
Family financial status (*N* = 487)	Very low (*N* = 19)	21.1	26.3	52.6	3.9	0.888
	Low (*N* = 59)	23.7	32.2	44.1	12.1	
	Moderate (*N* = 303)	23.1	26.4	50.5	62.2	
	High (*N* = 86)	18.6	27.9	53.5	17.7	
	Very high (*N* = 20)	30.0	15.0	55.0	4.1	

### Descriptive findings

Results about knowledge of harm (combined and individually), comparative harm, and risk perceptions are presented in Table 2 by smoking status. Of all respondents, 41.9% answered the three basic questions correctly, with only 21.6% of current smokers answering the three questions correctly. About 70% of respondents correctly answered each of three questions: that smoking shortens life and damages quality of life; that exposure to smoke from combustible cigarettes harms both heart and lungs; and that all or most heavy smokers suffer health problems due to their smoking. The question about the burden of mortality from smoking relative to traffic accidents led to the least-accurate answers: over 70% did not correctly answer this question. The same question also led to the highest percent (36.3%) of declarations of “don’t know” of any question. Current smokers’ responses were consistently the least accurate.

**Table 2:** Knowledge about tobacco harm and exposure among Israelis, for individual questions, by smoking status

		Current Smoker (%)	Former Smoker (%)	Never Smoker (%)	Overall (%)	N	*p*-value
Knowledge 1: Length and quality of life	Shortens life	5.4	9.0	7.9	7.6	38	
	Damages quality of life	17.1	9.7	9.1	11.0	55	
	Shortens life and damages quality	57.7	75.4	77.1	72.3	360	
	Neither	7.2	0	0.8	2.0	10	
	Don’t know	12.6	6.0	5.1	7.0	35	
	N	111	134	253		498	<.001
Knowledge 2: Secondhand smoke causes lung cancer and/or heart disease	Lung cancer	5.4	9.0	12.8	10.1	50	
	Heart disease	0.9	0.8	2.0	1.4	7	
	Both	57.7	73.1	73.2	69.7	345	
	Neither	9.0	5.2	2.0	4.4	22	
	Don’t know	27.0	11.9	10.0	14.3	71	
	N	111	134	250		495	<.001
Knowledge 3: Smoking in Israel kills less than/more than/same as are killed in traffic accidents	Less	36.9	17.0	21.6	23.8	118	
	More	19.8	29.6	32.0	28.6	142	
	Same	9.9	11.1	12.0	11.3	56	
	Don’t know	33.3	42.2	34.4	36.3	180	
	N	111	135	250		496	.008
Knowledge 4: How many heavy smokers suffer or will suffer from health problems because of their smoking?	Everyone	8.0	17.8	19.6	16.5	83	
	Most	47.3	51.9	54.9	52.4	263	
	Half	23.2	14.8	14.1	16.3	82	
	Minority	10.7	2.2	2.8	4.4	22	
	No one	1.8	0	0.8	0.8	4	
	Don’t know	8.9	13.3	7.8	9.6	48	
	N	112	135	255		502	<.001
Knowledge of harm (based on 3 questions [Knowledge 1, Knowledge 2, Knowledge 4])	3 questions not answered correctly	78.4	56.4	50.0	58.1	287	
	3 questions answered correctly	21.6	43.6	50.0	41.9	207	
	N	111	133	250		494	<.001

Across all respondents, the question with the highest associated risk perception was about the likelihood of affecting the health of the child who is present in a car in which smoking takes place (Mean:6.36; SD: 1.16, *n* = 487), and the question with the lowest associated risk perception concerned the severity of damage to adults’ health from smoking which occurs around them (Mean: 5.87; SD: 1.47; *N* = 470). The other two questions fell in the middle (Likelihood of an adult being harmed by tobacco smoke exposure: Mean 6.15; SD: 1.29; *N* = 486; Severity of harm to child exposed in a car: Mean: 6.09; SD: 1.41; *N* = 473). Overall, respondents perceived risk at a value of 24.5 (SD = 4.5) out of 28 possible points. Cronbach’s alpha for CRPS was 0.89, indicating that the items in the test are highly correlated.

### Univariable analyses

Univariable analyses of knowledge of harm, comparative knowledge, and risk perceptions are presented by population sector, gender, age category, smoking status, educational level, and family financial status in Table 3. Smoking status was significantly associated with knowledge of harm (*p* < .001), while the association with comparative harm approached statistical significance (*p* = .059). Just 21.6% of current smokers, but 50.0% of never-smokers, accurately answered all three questions about harm correctly. The percentage with correct answers among those with post-high school education was double the percentage among those who had not even attended high school (> 12 years: 49.8% correct; < 12 years: 25% correct, *p* < .001). Younger age groups were associated with higher levels of knowledge of harm. Male gender was associated with greater comparative knowledge (Males: 34.2%, Females: 23.1%, *p* = .007).

**Table 3:** Univariable analyses of knowledge and risk perceptions

		Knowledge of harm, 3 questions combined, % correct (*N* = 495)	*p*-value (Chi-squared except where indicated otherwise)	Knowledge of comparative harm, relative to traffic accidents, % correct (*N* = 497)	*p*-value (Chi-squared except where indicated otherwise)	Perception Scale Mean (SD) (*N* = 465)	*p*-value, N
Population Sector	Arab	38.6 (83)	0.483	27.4 (84)	0.791	26.1 (3.3) 84	<.001 464
	Jewish	42.7 (412)		28.8 (413)		24.2 (4.7) 380	
Gender	Male	38.5 (244)	0.120	34.2 (246)	0.007	24.4 (4.8) 221	0.455 465
	Female	45.4 (251)		23.1 (251)		24.7 (4.3) 244	
Age group	18–29	52.3 (88)	0.014	33.0 (88)	0.416	23.4 (4.8) 83	0.017 459
	30–49	45.6 (182)		25.1 (183)		24.3 (4.7) 173	
	50–65	39.7 (146)		31.8 (148)		25.2 (4.2) 136	
	> 65	28.4 (74)		26.0 (73)		25.2 (3.8) 67	
Educational level	< 12	25.0 (48)	< 0.001^a^	25.0 (48)	0.539^a^	24.7 (5.5) 45	0.551 462
	12	35.8 (190)		28.6 (189)		24.6 (4.5) 178	
	> 12	49.8 (253)		29.7 (256)		24.4 (4.5) 239	
Family financial status	Very low	27.8 (18	0.090^a^	27.8 (18)	0.127^a^	23.9 (4.2) 18	0.930 450
	Low	28.8(59)		18.6 (59)		24.7 (4.7) 53	
	Moderate	44.0(300)		29.3 (300)		24.5 (4.6) 281	
	High	48.8 (84)		37.2 (86)		24.6 (3.8) 80	
	Very high	40.0 (20)		25.0 (20)		23.9 (7.2) 18	
Smoking Status	Current	21.6 (111)	< 0.001	19.8 (111)	0.059	22.7 (6.0) 101	<.001 464
	Former	43.6 (133)		29.6 (135)		24.9 (4.0) 119	
	Never	50.0 (250)		32.0 (250)		25.1 (3.9) 244	

^a^Logistic regression with continuous explanatory variable.

Risk perception was significantly (*p* < 0.001) associated with smoking status. Current smokers had the lowest risk perceptions (Mean: 22.7, SD: 6.0), and never smokers the highest risk perceptions (Mean: 25.1, SD: 3.9). Those in the Arab sector reported higher perceptions of risk than those in the Jewish sector (Arab: Mean: 26.1, SD: 3.3; Jewish: Mean: 24.2, SD: 4.7, *p* < .001). Risk perceptions was associated with age group of the respondent (*p* = 0.017), with older respondents holding higher levels of risk perceptions.

### Multivariable models

Results of the multivariable statistical models are presented in Table 4.

**Table 4:** Multivariable statistical model results

Variable	Knowledge of 3 questions (Y/N) (N in model = 471)	*p*-value	Knowledge of comparative harm relative to traffic accidents (N in model = 473)	*p*-value	Risk perceptions of harm due to involuntary exposure to tobacco smoke (N in model = 441)	*p*-value
	Odds Ratio (Confidence Interval)		Odds Ratio (Confidence Interval)		Least Square Means	
Population Sector		0.188		0.668	Jewish: 23.7	< 0.001
Arab vs. Jewish	0.70 (0.41, 1.19)		0.89 (0.51, 1.54)		Arab: 26.1	
Gender		0.525		0.002	Male: 25.0	0.476
Female vs. Male	1.14 (0.76, 1.71)		0.50 (0.33, 0.77)		Female: 24.7	
Age group		0.015		0.539	65+:25.6	0.001
18–29 vs. 65+	3.01 (1.47, 6.19)		1.25 (0.60, 2.61)		18–29: 23.4	
30–49 vs. 65+	2.50 (1.29, 4.85)		1.00 (0.50, 1.99)		30–49: 24.8	
50–65 vs. 65+	1.78 (0.93, 3.43)		1.41 (0.73, 2.75)		50–65: 25.8	
Educational level	1.43 (1.05, 1.96)	0.025	1.11 (0.80, 1.53)	0.538	NR	0.309
Family financial status	0.83 (0.64, 1.07)	0.149	0.88 (0.67, 1.15)	0.347	NR	0.756
Smoking Status		< 0.001		0.017	Never: 25.8	< 0.001
Current vs. Never	0.28 (0.16, 0.49)		0.44 (0.25,0.78)		Current: 23.3	
Former vs. Never	0.93 (0.58, 1.48)		0.70 (0.43, 1.15)		Former: 25.5	

*NR* Not Relevant.

#### Knowledge of harm

Multivariable results were similar to findings from the univariable analyses: smoking status was significantly associated with knowledge of harm, with current smokers having the lowest levels and never smokers the highest levels (*p* < .001). Those in the younger age groups were better informed than those in the older age groups (*p* = .015), and those with higher educational levels were better informed (*p* = .025).

#### Knowledge of comparative harm

Multivariable results were similar to findings from univariable analyses. In both cases, current smokers were less informed, with a statistically significant result in the multivariable analyses (*p* = .017). Males were better informed relative to females (*p* = .002) in the multivariable as in the univariable analysis. Population sector, age group, educational sector, and family financial status were not significant, as in the univariable models.

#### Risk perceptions

Again, findings from the multivariable model closely followed those from the univariable models. Population sector was statistically significant, with Arabs holding higher risk perceptions than Jews (*p* < .001). Older individuals held higher risk perceptions (*p* = .001). Never smokers had the highest risk perceptions and current smokers the lowest (*p* < .001). Neither gender, educational level, nor family financial status were associated with perception levels.

#### Sensitivity analysis

For knowledge of harm, the multivariable analysis which included family income produced estimates which were in the same direction as the analysis using family financial status for all variables. (Note: The direction of the effect of family income and family financial status seemed to differ, but did not. Family financial status used descending order, while family income used ascending order.) When including family financial status in the model, educational level approached significance (*p* = .054), and higher family income was significantly associated with more accurate responses (*p* = .008). No differences in statistical significance (<.05 or > .05) were found for comparative knowledge or for risk perceptions between the multivariable models using family income and the models using family financial status as a proxy for socio-economic status.

## Discussion

The central findings of this study are that, 1) as a group, Israeli adults are only partially informed about the harms of tobacco smoking and exposure, and 2) smokers are particularly ignorant. Just over two in five respondents correctly answered three basic questions about knowledge of harm; among smokers, just over one in five respondents answered these three questions correctly. About 30% of respondents answered each of three of the knowledge questions incorrectly. Of particular interest was the comparative harm question which addressed numbers of deaths caused by smoking versus number of deaths caused by tobacco relative to traffic accidents. Over 70% of Israelis, and 80% of current smokers, were unaware that smoking causes more deaths than traffic accidents. Just over one-third of respondents incorrectly answered “less” or “same”, while over one-third (36.3%) answered, “I don’t know.” This occurred despite the fact that Israeli deaths due to smoking (approximately 8000) [21] are over twenty times greater than those due to traffic accidents (352) [22]. Risk perceptions were high overall, with an average of 24.5 (SD 4.5) out of a possible 28.

About 80% of Israelis knew that secondhand smoke exposure causes lung cancer, and about 70% knew that secondhand smoke exposure causes heart disease. Among smokers, these numbers were 63% and 59%, respectively. While this denotes ignorance among many respondents (particularly among smokers), international figures show that knowledge varies widely by country (Table 5) [9, 23–26], and is not universally high elsewhere, either. The causal effect of secondhand smoke on lung cancer in Canada, the UK, Australia, and the U.S. was understood by 79.6, 75.2, 69.0, and 68.1% of respondents, respectively, according to a 2006 report [27]. In a 2014 report about Bangladesh, 60% of respondents unexposed to tobacco smoke, and 40% of exposed respondents, were aware of this relationship [28]. Knowledge was reportedly lower in a pilot study in India published in 2012 (30–45%) [29], though not in China (nearly 60%), according to a 2010 report [26]. Knowledge that secondhand smoke causes cardiovascular disease in nonsmokers in India was poor, ranging from about 5% to under 40% [29].

**Table 5:** Knowledge about tobacco-attributable heart and lung disease risk in different countries

Study	Country	Respondents who believe that smoking causes	
		Lung cancer (%)	Heart Disease^a^ or Heart Attack^b^ (%)
Siahpush, 2006	USA	94.4	85.8^a^
	Canada	94.8	90.9^a^
	UK	93.7	89.6^a^
	Australia	94.3	88.6^a^
Yang, 2010	China	73	40.2^a^
Fakir, 2011	Morocco	63	22^a^
Agaku, 2014	29 African countries*	Median:5.1	Median:12.8^a^
	Tunisia	Lower limit: 0	Lower limit: 0^a^
	Somaliland, Somalia	Upper limit: 80.4	
	Malawi		Upper limit: 36.6^a^
Minh An, 2013	Vietnam	95.8	60.9^b^
Gupta, 2014	Thailand	97.4	75.2^b^
	Uruguay	96.9	92.3^b^
	Turkey	96.8	94.4^b^
	Mexico	96.6	80.5^b^
	Egypt	96	94.8^b^
	Brazil	96	87.1^b^
	Vietnam	95.2	63^b^
	Bangladesh	94.2	90.2^b^
	Poland	91.8	79.5^b^
	Philippines	91.5	77.6^b^
	Ukraine	89.7	75.5^b^
	Russian Federation	88.5	65.7^b^
	India	87.2	65.1^b^
	China	79	40.6^b^

*Reported median and range.

Consistent with previous reports, we found that knowledge and risk perceptions were lower among current smokers than among others. Previous research has shown that nonsmokers and former smokers viewed smoking as a riskier behavior than did current smokers [30]. Nonsmokers also viewed exposure to secondhand smoking as more risky, relative to smokers [18].

Previous research has repeatedly shown that other factors, in addition to smoking status, are associated with knowledge and risk perceptions. Within countries, educational level and socio-economic status are of particular importance. In studies conducted in the US, UK, Canada, Australia [9, 31], Bangladesh [32], and Vietnam [33], educational level has emerged as one of the strongest predictors of smoking knowledge. Knowledge and risk perceptions also increase with socioeconomic status (SES); this has been observed in the US, UK, Canada, Australia [9], and Morocco [24]. The International Tobacco Control study found that in the United States, Canada, United Kingdom, and Australia [9], the vast majority of respondents across SES strata and educational levels in these countries were able to answer some questions correctly. For example, the least-informed populations were poorly-educated Americans, of whom 83.7% knew smoking causes heart disease, and low-income British, of whom 90.5% knew that smoking causes lung cancer. Canadian respondents scored the highest, with the adjusted odds ratio (95%) from logistic regression being 1.61 (1.33–1.95) regarding heart disease, and 1.16 (0.89–1.52) regarding lung cancer [relative to the US population].

Other than for smoking status, we did not observe consistent patterns among the socio-demographic and economic variables on our three endpoints of harm, comparative harm, and risk perceptions. There were no differences between population sectors in knowledge of harm or comparative harm, though risk perceptions were slightly but significantly higher among Arabs than among Jews. Gender differences were apparent only for the knowledge of comparative harm endpoint; men were better informed than women about the deaths caused by tobacco relative to the deaths caused by traffic accidents. Respondents in younger age categories were better informed about harm relative to older respondents, but older respondents had higher risk perceptions. Those with higher education were better informed about harm, but not about comparative harm, than those with lower educational levels, and did not differ on risk perceptions. Family financial status was not significantly associated with knowledge of harm, knowledge of comparative harm, or risk perceptions. In the sensitivity analysis, we observed that family income was not associated with comparative knowledge or risk perceptions, but those with higher incomes did have higher levels of knowledge.

A recent report suggested that smoking rates in Israel have increased in recent years among young adults (2009: Past year: 37.6% Past month: 33.0%; 2016: Past year: 45.7%, Past month: 35.2%) [12]. This worrisome finding, along with the failure substantially to lower adult smoking prevalence – during a time of large reductions elsewhere, in countries such as the U.S. [34], Canada [35], and the UK [36] -- is a clear call to immediate action for tobacco control. Taxation is the strongest tobacco control tool readily available to policy-makers today. At present in Israel, taxation on roll-your-own (RYO) cigarettes is substantially less than that on packaged cigarettes, making RYO cigarettes particularly appealing to youth and socially disadvantaged individuals, and possibly discouraging quitting among smokers [37]. Raising taxes on RYO cigarettes is an important move which should be taken immediately; the issue is currently being discussed in the Supreme Court. Increasing the age at which tobacco can be legally sold from 18 to 21 is likewise a step of tremendous importance, particularly since roughly half of Israeli smokers began smoking between the ages of 18–21 [38]. That step would also assist the army in its battle for tobacco control. A complete advertising ban on all tobacco products, as required by Israel’s ratified agreement with the World Health Organization’s Framework Convention for Tobacco Control (FCTC), and also as stipulated in the governmentally-approved National Plan for Reduction in Smoking and its Damage, should be passed immediately, without any exemptions. Display bans, plain packaging, and graphic warnings are other important measures which have been enacted in many other countries and should be legislated in Israel. If passed, the legislation currently under discussion in the Economics Committee of the Knesset will contribute to bringing Israel back into the company of other progressive nations on tobacco control, though that bill is weakened by the exemption for advertising in the print press and the lack of required graphic warnings. In order to stay ahead of industry promotional efforts and decrease smoking prevalence, core funding for tobacco control is essential. Such a program should include funding for media campaigns such as the Truth Campaign [39] which has been so successful in the U.S., and strict enforcement of all tobacco control laws, including those related to youth access, advertising, and laws against smoking in public places. Laws to protect non-smokers from tobacco exposure, and Israel’s strong smoking cessation program, which is available to all through Israel’s comprehensive national health insurance system, should be strengthened. Systematic monitoring of behavior, knowledge, and attitudes of Israelis towards all nicotine and tobacco products, of tobacco and nicotine industry activity in Israel, and of the impact of tobacco industry activity and governmental policy on population level smoking prevalence, is crucial.

Because higher risk perceptions among smokers are associated with greater likelihood of making quit attempts and, to some extent, sustained smoking abstinence [40], the findings from this paper suggest that policies promoting public education may be of great importance. Public information campaigns could be conducted through traditional media channels (television and radio) as well as digital media and social media (for example, Facebook). Because smokers have the lowest levels of knowledge and risk perceptions, targeting smokers is particularly important. Graphic warnings on cigarette packs, as are used in over one hundred countries, have shown greater effectiveness in communicating risk, as have larger warnings and those with more content, than have smaller, non-graphic warnings [27]. Recent advances in product packaging, including package inserts and on-cigarette messaging (for example, “smoking kills” printed on cigarettes), are also promising and should be considered [27, 41, 42]. All of the product packaging and display approaches are directed at smokers and potential smokers, and so should be particularly efficient. In Israel, stronger package warnings, including graphic package warnings, inserts, and on-cigarette warnings, could regularly expose 1.3 million current smokers to these critical messages, in a very cost-efficient manner.

Understanding public knowledge and risk perceptions about combusted tobacco is especially important given the rapidly-changing tobacco landscape. New tobacco and nicotine products, including but not limited to electronic cigarettes and heated tobacco products, are emerging globally and in Israel. There are deep divisions within the professional community about the short and long-term effects of these new products on individuals and populations [43]. Industry sources claim substantial harm reduction from specific emerging nicotine and tobacco products, and employ such claims when promoting these new products to policy makers and the public [44]. To date, the government has not provided Israelis with clear information on these issues. To formulate realistic, effective policy, it is essential to understand public knowledge and risk perceptions regarding the dangers of the combustible cigarette, and use that information to give high priority to educating the public about all tobacco and nicotine products so that individuals can make wise choices.

### Strengths and limitations

The main strength of this study is that it was a nationally-representative study conducted in a broad, heterogeneous population, with a reasonably high response rate (61%). This allows generalization of the findings to be made to the Israeli population on an important topic – knowledge and risk perceptions of Israelis concerning smoking --which has not previously been studied in this population. That the study was conducted some time ago (December, 2010) means that it provides good baseline information about knowledge and risk perceptions about smoking, prior to the recent introduction of emerging tobacco and nicotine products, such as IQOS and JUUL [44, 45]. This is particularly important because, unlike in many other countries, such information is not available at all in the Israeli setting.

There are some methodological limitations. This was a landline-only survey. Because of the proliferation of cellphones in Israel, it is possible that some groups, particularly young people who owned cellphones but not landlines at the time of study, or people who owned landlines but did not use them frequently, are poorly-represented. Young adults who served in compulsory military service and lived in military housing (as opposed to returning home in the evenings) are likely under-represented. While the response rate was reasonable, we were unable to ascertain opinions of non-respondents.

The lack of standardized questions about knowledge and risk perceptions [46] limited our ability to make comparisons on all items. Question format may have affected participants’ answers [47], and differences in methods pose a statistical challenge for comparison between studies. It is imperative to create a standard set of questions and answers to measure and compare a population’s knowledge of the health effects of smoking.

Because this was not a very large dataset, we choose to focus our analyses on variables of greatest interest. Other variables, both measured and unmeasured, may have acted as confounders.

As is done in the great majority of population studies both in Israel and internationally about smoking, smoker status was based on self-report. Self-report has been validated as a measure of smoking status [48]. In this study, we obtained the information through telephone interviews. Self-report in the context of telephone interviews have been shown to under-estimate smoking status by 3–4% [49]. Therefore, it is reasonable to assume that a small percentage of smokers may not have identified as smokers, resulting in misclassification bias of a small percentage of respondents.

## Conclusions

Like many others, Israelis do not fully grasp tobacco’s true dangers. Effective communication of the full range of tobacco risks to the public, with targeting of smokers, is an essential component of comprehensive tobacco control policy.

## Additional file

Additional file 1: Annotated Questionnaire. Sensitivity analysis: Multi-variable statistical model results, using family income instead of family financial status. (DOCX 43 kb)
