# Supplementary material for: Knowledge and risk perceptions of Israelis towards combustible cigarettes: the need for immediate remedial action
Source: Isr J Health Policy Res. 2019 Jan 14;8:10. doi: 10.1186/s13584-018-0276-2 (PMC6330745; doi:10.1186/s13584-018-0276-2)
Supplement: Supplementary file 1 — Annotated Questionnaire. Sensitivity analysis: Multi-variable statistical model results, using family income instead of family financial status. (DOCX 43 kb) [file 13584_2018_276_MOESM1_ESM.docx]

Additional file 1 **Annotated Questionnaire**

PUBLIC OPINION SURVEY: SMOKING AND GOVERNMENT

(SELECTED QUESTIONS)

Hello. My name is ___ and I am speaking with you from the Cohen Research Institute of Tel Aviv University in the context of a national survey which deals with health and smoking. The survey is being conducted by investigators from Tel Aviv University and Bar Ilan University, with funding from the National Institute for Health Policy Research. Your opinions are important to us and we thank you for your participation in the survey. We guarantee that your responses will be confidential and used for study purposes only.

1. AGE How old are you? _______ 99 Refuse [Interview age 18 and above]
2. AGEGROUP For nonresponders: To what agegroup do you belong?
   1. 18-21
   2. 22-29
   3. 30-39
   4. 40-49
   5. 50-59
   6. 60-65
   7. 65+
   8. [Don't read:] Refuse
3. SEX Sex
   1. Male
   2. Female
4. EDUC Where did you last study?
   1. I didn't study at all
   2. Elementary school or Junior High
   3. Highschool (IYUNI/ professional/agricultural/ technological / yeshiva)
   4. A certificate granting institution (up to one year)
   5. College/ Seminary / Post high school Yeshiva
   6. Academic institution
5. [Don't read :] Refuse

Q31.To the best of your knowledge, what is the likelihood that a child who travels regularly in a car where people are smoking will be hurt? Please answer on a 1-7 scale, where 1 means the likelihood is very low and 7 means the likelihood is very high.

| Highly likely |  |  |  |  |  | Highly unlikely | Don't know/ Refuse |
| --- | --- | --- | --- | --- | --- | --- | --- |
| 7 | 6 | 5 | 4 | 3 | 2 | 1 | 9 |

Q32. And, to the best of your knowledge, what is the likelihood that an adult will be hurt who is regularly exposed to other people's smoking? Please answer on a 1-7 scale, where 1 means the likelihood is very low and 7 means the likelihood is very high.

| Highly likely |  |  |  |  |  | Highly unlikely | Don't know/ Refuse |
| --- | --- | --- | --- | --- | --- | --- | --- |
| 7 | 6 | 5 | 4 | 3 | 2 | 1 | 9 |

Q33.To the best of your knowledge, how severe will the damage be to a child who travels regularly in a car where people are smoking? Please answer on a 1-7 scale, where 1 means minimal or no damage and 7 very severe damage.

| Severe |  |  |  |  |  | Minimal or none | Don't know/ Refuse |
| --- | --- | --- | --- | --- | --- | --- | --- |
| 7 | 6 | 5 | 4 | 3 | 2 | 1 | 9 |

Q34.To the best of your knowledge, how severe will the damage be to an adult who is regularly exposed to other people's smoking will be hurt? Please answer on a 1-7 scale, where 1 means minimal damage or no and 7 very severe damage.

| Severe |  |  |  |  |  | Minimal or none | Don't know/ Refuse |
| --- | --- | --- | --- | --- | --- | --- | --- |
| 7 | 6 | 5 | 4 | 3 | 2 | 1 | 9 |

Q35. To the best of your knowledge, people who smoke regularly are apt to: [Read in random order]

(Correct answer: 3)

1. Shorten their life
2. Injure their quality of life
3. Shorten their life and injure their quality of life
4. Neither
5. Don't know

9. [Don't read] Refuse to answer

Q36. To the best of your knowledge, does regular passive smoking increase the chances of heart disease or lung cancer?

(Correct answer: 3)

1. Yes, lung cancer but not heart disease
2. Yes, heart disease but not lung cancer
3. Yes, both heart disease and l ung cancer
4. Not heart disease or lung cancer

8. Don't know

9. [Don't read] Refuse to answer

Q37. To the best of your knowledge, smoking in Israel causes :

(Correct answer: 2)

- 1. Less deaths than traffic accidents
  2. More deaths than traffic accidents
  3. The same number as are killed in traffic accidents

8. Don't know

9. [Don't read] Refuse to answer

Q38. To the best of your knowledge, how many **heavy** smokers suffer or will suffer from health problems, due to their smoking?

(Correct answer: 1 or 2)

1. All of them

2. Most of them

3. About half of them

4. A minority of them

5. No one

8. Don't know

**And to finish up, a few personal questions for statistical purposes:**

Q39. Do you smoke?

1. Yes, every day

2. Yes, sometimes

3. No, but I used to smoke every day

4. No, but I used to smoke sometimes

5. No, I never smoked

9. [Don't read] Refuse to answer

Q43. Are you

1. Jewish

2. Muslim

3. Druze

4. Christian

7. Other

9. [Don't read] Refuse to answer

Q46. Where were you born?

1. Israel

2. USSR

7. Other ______Q46a Specify

8. [Don't read] Don't know

9. [Don't read] Refuse to answer

Q49. FAMILY FINANCIAL STATUS
How would you rate the financial status of your family?

1. Very high

2. High

3. Moderate

4. Low

5. Very low

9. [Don't read] Refuse to answer

Q50. FAMILY INCOME
The average family income in Israel today is about NIS 11,000 neto. Is your family income

1. Much below average

2. A little below average

3. Average

4. A little above average

5. Way above average

6. Not relevant, Kibbutznik

9. Refuse to answer

**THANK YOU FOR YOUR PARTICIPATION
For administrative purposes**

Q54. Language of interview

1. Hebrew
2. Arabic
3. Russian

Sensitivity analysis: Multi-variable statistical model results, using FAMILY INCOME instead of FAMILY FINANCIAL STATUS

|  | Knowledge of 3 questions (Y/N)  (N in model = 439) |  | Knowledge of comparative harm relative to traffic accidents (N in model = 440) |  | Risk perceptions of harm due to involuntary exposure to tobacco smoke (N in model = 410) |  |
| --- | --- | --- | --- | --- | --- | --- |
| Variable | Odds Ratio (Confidence Interval) | p-value | Odds Ratio (Confidence Interval) | p-value | Least Square Means | p-value |
| Population Sector |  | 0.303 |  | 0.892 | Jewish: 24.1 | .003 |
| Arab vs. Jewish | 0.75 (0.43, 1.30) |  | 0.96 (0.55, 1.69) |  | Arab: 26.0 |  |
| Gender |  | 0.430 |  | 0.001 | Male: 25.2 | 0.453 |
| Female vs. Male | 1.19 (0.78, 1.82) |  | 0.48 (0.31, 0.75) |  | Female: 24.9 |  |
| Age group |  | 0.023 |  | 0.604 | 65+: 25.8 | 0.008 |
| 18-29 vs. 65+ | 3.11 (1.46, 6.60) |  | 1.28 (0.60 2.74) |  | 18-29: 23.8 |  |
| 30-49 vs. 65+ | 2.31 (1.16, 4.60) |  | 0.95 (0.47, 1.91) |  | 30-49: 24.8 |  |
| 50-65 vs. 65+ | 1.74 (0.89, 3.43) |  | 1.29 (0.66, 2.55) |  | 50-65: 25.8 |  |
| Educational level | 1.39 (0.99, 1.94) | 0.054 | 1.11 (0.79, 1.55) | 0.558 | NR | 0.104 |
| Family income | 1.26 (1.06, 1.49) | 0.008 | 1.03 (0.87, 1.23) | 0.717 | NR | 0.180 |
| Smoking Status |  | <0.001 |  | 0.048 | Never: 25.8 | .001 |
| Current vs. Never | 0.28 (0.16, 0.50) |  | 0.49 (0.27, 0.87) |  | Current: 23.8 |  |
| Former vs. Never | 0.95 (0.58, 1.55) |  | 0.72 (0.43, 1.20) |  | Former: 25.6 |  |
